# Nutrition literacy across adolescence stages in Egypt: a quartile-based analysis for tailored educational strategies

**DOI:** 10.1186/s12889-025-23583-6

**Published:** 2025-07-05

**Authors:** Iman H. Kamel, Ammal M. Metwally, Dina A. Zaki, Raefa Refaat Alam, Amani E. Ali, Ghada A. Elshaarawy, Safaa I. Abd El Hady, Ahmed G. Samy, Mona A. Elabd

**Affiliations:** 1 Child Health Department, Medical Research and Clinical Studies Institute, National Research Centre (Affiliation ID: 60014618), Dokki, Cairo Egypt; 2 Health and Community Medicine,Community Medicine Research Department, Medical Research and Clinical Studies Institute, National Research Centre (Affiliation ID: 60014618), P.O. 12622, Dokki, 60014618 Cairo Egypt; 3 Faculty of Nursing, Mansoura National University, Al Dakhlyia, Egypt; 4 Technical Institute of Nursing, Sherbin, Al Dakhlyia, Egypt; 5 Department of Psychiatry, Mansoura General Hospital, Ministry of Health and Population, El Dakahlyia, Egypt; 6 Biological Anthropology Department/ Medical Research and Clinical Studies Institute, National Research Centre (Affiliation ID: 60014618), Dokki, Cairo Egypt

**Keywords:** Nutrition literacy (NL), Total nutrition literacy (TNL), Critical nutrition literacy (CNL), Functional nutrition literacy (FNL), Interactive nutrition literacy (INL), Adolescence stage, Body mass index (BMI), Family dynamics, Vitamin and mineral intake, Egyptian adolescents

## Abstract

**Background:**

Low nutrition literacy (NL) among adolescents can worsen health and economic inequalities, potentially leading to a higher burden of non-communicable diseases.

**Aim:**

This study aimed to assess Total Nutrition Literacy (TNL) and its domains across different adolescent stages with an in-depth look at NL among Egyptian adolescents, through a quartile-based approach. It analysed the relation between adequate NL influences and vitamin, mineral intakes and body mass index (BMI) categories (underweight, normal, overweight/obese). It explored the effects of adolescent characteristics (gender, BMI) and family environment (parental education, food literacy, health status) on achieving adequate TNL levels.

**Methods:**

A cross-sectional study was conducted with 1,050 Egyptian adolescents aged 10–19 from various geographical and socioeconomic backgrounds. Data were collected using a self-administered questionnaire measuring demographic information, TNL and its domains (categories) (Functional (FNL), Interactive (INL), and Critical (CNL)), and parental food literacy (PFL). Anthropometric measurements assessed nutritional status via BMI. Statistical analyses using SPSS identified associations and predictors of adequate TNL.

**Results:**

TNL scores rose significantly from 68.8 in early adolescence (10–13 years) to 77.4 in late adolescence (17–19 years) (*p* < 0.001). All domains improved with age (*p* < 0.001), with females outperforming males in INL and CNL (*p* < 0.001). FNL level was notably low at 56.6%, compared to higher rates in INL (84.1%) and CNL (91.0%). Meanwhile, the median scores for NL remain relatively close to the lower levels of adequacy across all stages. Parental employment, PFL, health status, and family dynamics were significant predictors of adequate TNL. Maternal education became particularly crucial in late adolescence, while PFL had the greatest influence in early and middle adolescence. Adequate TNL increased the likelihood of reporting vitamin intake by nearly five times (COR = 4.9, *p* < 0.01). The relation between TNL and its domains with BMI vary across adolescence.

**Conclusion:**

Using quartile distribution to assess literacy adequacy provided a detailed view of literacy gaps, allowing identification of adolescents most in need of intervention. NL programs have to be tailored to meet adolescents’ evolving needs, emphasizing foundational literacy in early adolescence, interactive skills in middle adolescence, and critical literacy in late adolescence.

**Supplementary Information:**

The online version contains supplementary material available at 10.1186/s12889-025-23583-6.

## Introduction

Achieving “zero hunger” and reducing malnutrition are integral goals of the 2030 Sustainable Development Goals (SDGs), emphasizing food security and sustainable agriculture [1, 2]. However, food and nutrition crises in low- and middle-income countries, including Egypt, have intensified due to rising food prices and supply chain challenges [3]. In recent years, staple food costs in Egypt have increased by approximately 40%, driven by economic instability and global disruptions, leading families to rely on cheaper, nutrient-poor foods. This trend exacerbates health issues, such as obesity and nutrient deficiencies, particularly among children and adolescents [4, 5]. Currently, malnutrition affects 7.8% of children in Egypt through stunting, 8% through obesity, and 25% through anemia, reflecting the broader global trend where over 30% of children are stunted and 5.7% are overweight [6, 7]. Children from low-income backgrounds in the Middle East and North Africa (MENA) region face higher risks of poor diets and malnutrition, highlighting the need for effective interventions during adolescence, a stage of rapid growth and increased nutrient needs as defined by the World Health Organization (WHO) [8–10].

In Egypt, adolescents face significant nutritional challenges. A recent UNICEF report indicates that 25% of Egyptian adolescents are anaemic and 8% are obese, reflecting the dual burden of undernutrition and overnutrition [11]. Their dietary patterns are often characterized by high consumption of energy-dense, nutrient-poor foods, accompanied by inadequate intake of fruits and vegetables. Additionally, physical inactivity is widespread and contributes to the increasing prevalence of obesity among youth [12, 13]. National data further reveal that a substantial proportion of Egyptian adolescents do not meet recommended daily intakes for fruits, vegetables, and dairy products, while a significant percentage regularly consume fast food and sugar-sweetened beverages [14]. Furthermore, a regional scoping review on health literacy highlights that adolescents and young adults in the Eastern Mediterranean exhibit limited health literacy, particularly regarding nutrition and the prevention of non-communicable diseases [15]. This low level of health literacy impairs adolescents’ ability to interpret and apply nutrition-related information, thereby limiting their capacity to make informed dietary decisions [15, 16]. Despite these concerns, there remains a marked gap in research addressing nutrition literacy specifically among Egyptian adolescents, and few existing interventions are tailored to their developmental stage and contextual needs [17].

Improving adolescent nutrition literacy (NL)—the ability to access, comprehend, and apply nutritional information—has become crucial to empower informed dietary choices and reduce long-term health risks. Total Nutrition Literacy (TNL) includes Functional (FNL), Interactive (INL), and Critical (CNL) domains, each addressing different aspects of understanding and applying nutrition knowledge. FNL involves basic understanding, INL emphasizes communication and information-seeking, and CNL involves evaluating and advocating for improved nutrition policies [18–20]. The connection between NL and positive health outcomes is well-documented, with improved nutrition knowledge correlating with healthier dietary behaviors that support growth and reduce conditions like stunting and anemia [21, 22]. Yet, despite this understanding, comprehensive policies for promoting NL are lacking in the MENA region, including Egypt, where research on adolescent NL remains scarce. A recent study revealed that 18.1% of Egyptian adolescents exhibited inadequate NL, though limitations such as a small sample size suggest the need for broader studies [23–26].

Adolescence is a dynamic period characterized by distinct developmental stages—early (10–14 years), middle (15–17 years), and late (18–19 years)—each with unique physiological, cognitive, and social changes that influence nutritional needs and behaviors [27, 28]. Understanding these differences is essential for designing effective, stage-specific interventions. Early adolescence involves the onset of puberty, leading to rapid physical growth and increased nutrient requirements, with adolescents remaining heavily reliant on family and school environments for nutritional guidance [29, 30]. In middle adolescence, teenagers experience continued growth while gaining independence in their food choices, with peer influence playing a significant role and potentially leading to risk-taking behaviors that impact dietary habits [31, 32]. Late adolescence marks the transition to adulthood, where individuals gain greater autonomy over their diets, often influenced by major life changes such as entering the workforce or higher education [33, 34]. By examining TNL across these stages, this study aims to identify critical periods for intervention, ensuring that nutrition education strategies align with adolescents’ developmental needs. Early adolescence may be optimal for foundational education, middle adolescence for peer-driven initiatives and decision-making skills, and late adolescence for practical applications of nutrition knowledge in independent living contexts [35, 36].

This study aimed to address existing gaps by investigating the progression of TNL across the three stages of adolescence—early, middle, and late—among Egyptian adolescents. Specifically, it assessed the adequacy of Functional, Interactive, and Critical Nutrition Literacy at each developmental stage and analyzed its relationship with Body Mass Index (BMI) categories, including underweight, normal weight, and overweight/obese classifications. Additionally, the study examined how TNL adequacy influences vitamin and mineral intake, as well as overall dietary practices. Furthermore, it explored the role of individual characteristics such as age, gender, and BMI, along with family-related factors including parental education, food literacy, employment, and health status, in shaping adolescent TNL. These findings are anticipated to inform age-specific interventions that support adolescents in making healthier choices and achieving optimal nutritional status, ultimately aiding in pursuing SDG targets on health and food security [37, 38]. Specifically, the insights gained can guide the development of nutrition education programs in schools, community health initiatives, and parental engagement strategies, ultimately fostering a more health-conscious generation. Additionally, the study has implications for policymakers, highlighting the urgent need for integrating comprehensive nutrition literacy curricula into public health and educational frameworks [39].

## Subjects and methods

### Study design and target groups

A cross-sectional study was conducted across Egypt’s distinct geographical and socioeconomic regions from January to September 2022. The study targeted adolescents of both genders, aged 10–19 years, who consented to participate. The participants were categorized according to the World Health Organization (WHO) criteria for the stages of adolescence [40], early adolescence (10–13 years), middle adolescence (14–16 years), and late adolescence (17–19 years). The study aimed to assess the nutritional literacy of adolescents across these age groups.

### Sample size calculation and selection

The sample size was calculated based on an estimated proportion of 18.1% for adolescents with inadequate total nutrition literacy (TNL) in each adolescence stage. A sample size of 297 provided a two-sided 97% confidence interval with a margin of error of 0.100. The sample size was calculated as follows [41].

### Numeric results for Two-Sided confidence intervals for one proportion

**Table:** Confidence interval formula: exact (Clopper-Pearson)

Confidence Level	Sample Size (N)	Target Width	Actual Width	Proportion (P)	Lower Limit	Upper Limit	Width if *P* = 0.5
0.950	950	0.050	0.050	0.181	0.157	0.207	0.065

To ensure representation across the three stages of adolescence and socioeconomic strata, the sample size was increased to 1,050 participants, with 350 adolescents from each age group (early, middle, and late adolescence) [42]. By focusing on adolescents, the study captures a critical period of development during which nutrition literacy can significantly impact both immediate and long-term health outcomes.

### Study setting and participant selection

Participants were recruited from households through a **multi-stage random sampling** approach. Stage one was the selection of governorates to represent the main districts of four Egyptian regions. each governorate represented distinct geographical and dietary regions of Egypt: **Cairo** (representative of the Greater Cairo region representing **Urban/metropolitan region**); **Fayoum** (representative of Upper Egypt representing Agricultural region); **Al Dakhlyia** (representative of the Delta region) and **Marsa Matrouh** (representative of a border/frontier governorate). Each region has unique dietary habits and crops, contributing to the study’s focus on the diversity of dietary patterns across Egypt. These governorates were randomly chosen to capture various dietary practices, socioeconomic backgrounds, and cultural influences on nutrition. This selection enhances the study’s relevance to Egypt’s broader context [43, 44]. Cairo is a dense urban setting with high dietary diversity, while Fayoum and Al Dakhlyia represent agricultural areas with traditional dietary practices, and Marsa Matrouh, a frontier region, has limited food access compared to other areas.

For each governorate, both urban and rural areas were targeted for addressing the regional variability in nutrition literacy. By including both urban and rural households in each governorate, this study accounts for these disparities, enhancing the generalizability of the findings. During phase three, participants were stratified by socioeconomic status (SES) using the Economic Research Forum and CAPMAS (Central Agency for Public Mobilization and Statistics) wealth index (low, middle, and high). To minimize selection bias, random sampling was conducted in urban and rural districts within each SES group, with three cities and three local village units chosen per stratum. This structured approach also addresses potential oversampling biases in urbanized areas, ensuring that rural adolescent voices are represented adequately [45]. This selection was designed as part of a broader effort to identify children at high risk for autism, and it adhered to the study’s inclusion and exclusion criteria [46]. Adolescents were randomly selected through a community house-to-house approach.

For each targeted governorate, 45 participants (15 per adolescent stage) were recruited from each social class, resulting in a total of 225 adolescents from each governorate, with the exception of the Cairo governorate, which had 270 participants (90 per each social class, 30 per each adolescent stage).

### Inclusion criteria

The study included adolescents aged 10–19 years, classified according to the World Health Organization (WHO) adolescence stages, encompassing early, middle, and late adolescence. Both male and female participants were eligible, provided they had been residing in the selected governorates for at least one year to ensure their dietary habits reflected the local environment. To account for variations in educational background, the study included adolescents actively attending school in public, private, or community-based educational institutions, as well as those who had dropped out of school but had completed at least six years of formal education, ensuring they possessed the necessary literacy skills to engage with the study materials. Additionally, informed parental consent and adolescent assent were mandatory for participation.

### Exclusion criteria

Adolescents were excluded from the study if they had diagnosed cognitive impairments or severe learning disabilities that could hinder their ability to complete the nutrition literacy assessment. Those who had not completed at least six years of formal education were also excluded, as they might lack the foundational literacy skills required for the questionnaire To minimize confounding factors, adolescents with chronic medical conditions affecting nutrition intake, such as diagnosed eating disorders or metabolic disorders, were excluded unless their condition was a specific focus of the study. Additionally, non-Egyptian adolescents or those who had moved to Egypt within the past year were not included, as their dietary habits and environmental influences might not align with the local context. To prevent potential clustering biases within families, only one adolescent per household was selected for participation.

### Data collection instruments and procedures

#### Questionnaire administration

A self-administered questionnaire was utilized to gather data from the adolescent participants. This questionnaire was filled out under the guidance of the research team to ensure clarity and accuracy. The questionnaire was adapted from a previously validated tool, originally designed and published by Hoteit and colleagues [47], ensuring its relevance to the adolescent population in the context of nutrition literacy (NL) assessment. A pilot study involving 10% of the participants was conducted before the main study to enhance clarity, minimize ambiguity, and address potential sources of measurement error.

The questionnaire was divided into two major sections:

### Demographic and socioeconomic information

This section focused on collecting essential background information on the enrolled adolescents. The demographic variables collected included the participants’ **age**, **gender**, **educational level**, and details on their **primary caregiver** (i.e., who was primarily responsible for their daily care). Additionally, the study gathered data on the **education levels of both parents**, as parental education often influences the nutritional habits and literacy of children. Household Crowding Index: Calculated as the number of co-residents (excluding newborns) divided by the number of rooms (excluding kitchens and bathrooms [48–50].

Parents provided self-reported anthropometric data (weight and height) to calculate Body Mass Index (BMI), facilitating assessment of nutritional status in line with WHO’s BMI-for-age guidelines. Participants were instructed to provide recent height and weight measurements to reduce potential reporting biases, particularly in anthropometric data. However, the reliance on self-reported anthropometrics remains a limitation, as it introduces the possibility of data inaccuracy, an issue common in large-scale, self-administered surveys Participants also reported on their intake of **vitamins and minerals**, providing insight into their dietary supplementation habits.

### Vitamins assessment

Assessing adolescents’ consumption of dietary supplements focused on participants’ report on vitamins D, C, A, B12, and folic acid intake. Assessment of these particular vitamins was crucial due to the essential roles these micronutrients play during this critical developmental stage. Adolescence is marked by rapid growth and physiological changes, increasing the demand for nutrients that support bone development, immune function, cognitive maturation, and overall health.​ Monitoring supplement intake in this demographic helps identify nutritional gaps and informs interventions aimed at promoting balanced diets rich in essential vitamins.

#### Vitamin D

is vital for calcium absorption and bone mineralization, processes that are foundational during the adolescent growth spurt. Adequate vitamin D levels are necessary to achieve optimal bone density, reducing the risk of osteoporosis and fractures later in life [51].​.

#### Vitamin C

serves as a potent antioxidant and is essential for collagen synthesis, which is integral to the structural integrity of skin, blood vessels, and connective tissues. It also enhances immune defense mechanisms, aiding in the prevention and recovery from infections [52, 53].​.

#### Vitamin A

is crucial for vision, immune competence, and cellular differentiation. During adolescence, sufficient vitamin A intake supports the development of epithelial tissues and bolsters the body’s ability to combat pathogens [54].

#### Vitamin B12

is indispensable for neurological function and the formation of red blood cells. Its role in DNA synthesis and myelination of nerve fibers is particularly pertinent during adolescence, a period characterized by significant cognitive and physical development [55].​.

#### Folic acid (vitamin B9)

is essential for DNA synthesis and repair, supporting rapid cell division and growth. Adequate folic acid intake is vital during adolescence to prevent megaloblastic anemia and to support neural development [56].

### Minerals assessment

Understanding which supplements adolescents consume provides insights into existing nutrient gaps and overall dietary patterns. Dietary supplements, such as multivitamin/mineral products, have been shown to help fill nutrient gaps and improve micronutrient sufficiency among children and adolescents. However, there is a concern about the over-reliance on supplements as substitutes for whole foods, which can lead to lower overall energy intake and lack of consumption of other critical nutrients found in whole foods [57, 58]. The current study focused on participants’ report on consumption of dietary supplements, specifically calcium, magnesium, iron, and zinc, that is grounded in their critical role in growth, development, and overall health during this life stage [59]. Adolescence is a period of rapid skeletal growth and bone mineralization, making **calcium and magnesium** essential for maintaining strong bones and preventing future conditions like osteoporosis and fractures. Since bone mass peaks during adolescence, ensuring adequate intake of these minerals is crucial for long-term musculoskeletal health [60, 61].

Beyond skeletal development, iron and zinc are fundamental for cognitive function, immune health, and metabolic processes. **Iron** deficiency is a leading cause of anemia among adolescents, particularly in females due to increased iron loss from menstruation, which can lead to fatigue, decreased concentration, and poor academic performance [62]. Similarly, **zinc** plays a key role in immune function, wound healing, and enzymatic reactions, helping adolescents maintain overall health and fight infections during a stage of high physiological demand [63].

In this study, data on vitamin and mineral intake reflect self-reported use of dietary supplements only, specifically including calcium, magnesium, iron, zinc, and multivitamin preparations. These data do not include intake from food sources. Dietary intake of vitamins and minerals from regular meals was assessed as part of a broader project; however, those findings are presented in a separate manuscript.

### Nutrition literacy and food literacy assessment

The second part of the questionnaire measured the **nutrition literacy (NL)** of the adolescents and the **food literacy** of their parents. Nutrition literacy refers to the ability to obtain, process, and understand basic nutrition information needed to make appropriate health decisions.

To assess NL, the **Adolescent Nutrition Literacy Scale (ANLS)** developed by Bari [64], was utilized. This comprehensive tool consists of **22 questions**, **categorized** into three distinct **components**:

- **Functional Nutrition Literacy (FNL)**: This component, composed of **7 questions*assessing basic nutritional information comprehension***. It evaluated the adolescents’ ability to comprehend and use basic nutritional information, such as their understanding of nutrition-related scientific terms, dietary guidelines, and the recommendations provided by public health professionals. For instance, it included questions assessing participants’ familiarity with international dietary guidelines, such as those from the **World Health Organization (WHO)** regarding fruit and vegetable intake. The scoring range for FNL is **7–35**, with a cut off score of **≥ 21** indicating adequate functional literacy.
- **Interactive Nutrition Literacy (INL)**: The **6 questions*in this component measured the adolescents’ skills in seeking out***,*** discussing***,*** and applying nutrition-related information***within social contexts, including communication with peers, family members, and health professionals. The ability to engage with nutrition topics and translate this knowledge into practical actions, such as modifying dietary habits based on newly acquired information, was a key aspect of this component. The score for INL ranges from **6 to 30**, with a cut off score of **≥ 18** considered adequate.
- **Critical Nutrition Literacy (CNL)**: ***The****9 questions****in this section focused on the adolescents’ ability to critically assess nutrition information and influence***others’ dietary practices. It assessed participants’ engagement in activities that promote healthy eating, support for policies that improve dietary habits, and their ability to evaluate the credibility of nutrition-related information, particularly from social media and other sources. The score range for CNL is **9–45**, with a cut off score of **≥ 27** indicative of sufficient critical literacy.

#### Total nutrition literacy (TNL)

was calculated as the sum of the three components (FNL, INL, CNL), yielding a total possible score between **22 and 110**. A cut off score of **≥ 66** reflected adequate overall nutrition literacy. This metric provided a comprehensive view of the adolescents’ ability to understand, interact with, and critically evaluate nutrition information.

To assess **parental food literacy**, the validated **Short Food Literacy Questionnaire (SFLQ)** developed by Gréa Krause et al. [65] was used. The parental food literacy questionnaire was composed of **12 questions**, divided across three dimensions similar to those assessed in the adolescent scale but with fewer questions per category: **Functional food literacy** (6 questions); **Interactive food literacy** (2 questions); **Critical food literacy** (4 questions).

**The parental food literacy score** ranged from **7 to 52**, with a cut off score of **≥ 36** indicating adequate food literacy. This allowed for comparison between the literacy levels of parents and their children, providing a deeper understanding of family dynamics regarding nutrition knowledge and behaviors.

### Nutritional and growth status assessment

In addition to the self-reported data of the parents, a physical assessment of nutritional status was conducted. By conducting in-person measurements, this study enhances data reliability and consistency across rural and urban participants. Additionally, anthropometric data allows for exploring relationships between growth status and nutrition literacy, which could reveal developmental implications of inadequate nutrition literacy during adolescence. Anthropometric measurements of **weight** and **height** were taken using standardized equipment and techniques. **Weight** was measured with a **Seca Scale Balance**, while **height** was recorded using a **Holtain portable anthropometer**. These measurements were critical for evaluating the **growth status** of the adolescents, as weight and height are primary indicators of nutritional health. The BMI was calculated as weight (in kilograms) divided by height (in meters) squared based on the WHO growth standards with the help of the Anthro-Program of PC [66]. The body mass index (BMI) was evaluated as follows: underweight if BMI is less than 18.5, normal/healthy weight if BMI is 18.5 to 24.9, overweight is BMI is 25.0 to 29.9, and obese if BMI is 30.0 or higher [67]. The BMI classification provided an additional layer of insight into the participants’ nutritional health, correlating with their dietary habits and nutrition literacy levels.

### Measures to ensure validity and reliability of tools used for NL and FL assessment

Both **ANLS that is** developed by Bari [64] and SFLQ that is developed by Gréa Krause et al. [65] have been translated, culturally adapted and utilized in Arabic-speaking contexts. They have been adapted in a study to assess the nutrition literacy of adolescents across countries including Lebanon, Bahrain, Egypt, Jordan, Kuwait, Morocco, Palestine, Qatar, Saudi Arabia, and the United Arab Emirates [68]. The study involved 5,401 adolescent-parent dyads and found that 28% of adolescents had poor nutrition literacy. However, the study did not detail the process of translating or validating the ANLS and SFLQ for each specific Arabic-speaking context and it did not provide specific psychometric properties of the Arabic-translated tools.

We have conducted a multistep process to mitigate this for ensuring the tools appropriateness for our target population. Initially, a **pilot test** was conducted before large-scale implementation to ensure the clarity and appropriateness of the translated Arabic **ANLS** and **SFLQ tools.** This step involved administer of the Arabic **versions of the tools for 10% of different participants as a pilot sample** (*n* = 105) to assess their usability and ensure that participants could complete the questionnaire without difficulty. Subsequently, to assess internal consistency, the study employed Cronbach’s alpha [69]. with a larger sample of 330 participants, achieving high values of **Cronbach’s alpha (**0.89 for ANLS and 0.86 for SFLQ, ≥ 0.8) that indicated strong reliability [70]. To assess the stability of responses over time, a subset of 105 participants completes the Arabic SFLQ twice, with a two-week interval (**test-retest reliability)**. The Intraclass Correlation Coefficient (ICC) was calculated to measure consistency, achieving ICC of 94% and 92% respectively indicating excellent reliability (ICC ≥ 0.75) [71]. This comprehensive approach ensured that the Arabic ANLS and SFLQ are scientifically sound and culturally relevant tool for assessing nutrition literacy among Arabic-speaking adolescents.

### Statistical analysis

Data were analyzed using the **Statistical Package for Social Sciences (SPSS)**, version 26. Various statistical techniques were employed to summarize and analyze the collected data: **Categorical variables** (e.g., gender, social class, nutritional literacy categories) were summarized as **numbers** and **percentages**. **Continuous variables** (e.g., BMI, literacy scores) were presented as **means** and **standard deviations**.

Statistical significance was determined using: **Pearson’s Chi-square test (χ²)** and **Fisher’s exact test** to assess associations between categorical variables. **Z-tests** were applied for comparisons of proportions.For comparisons of means between groups, the **t-test** and **ANOVA** were utilized. Crude O**dds Ratio (COR)** with **95% confidence intervals (CI)** were calculated to examine associations between adolescence stages and nutritional literacy. **Logistic regression analysis** was conducted to identify significant predictors of **adequate TNL** among the adolescents.A ***p***-value < 0.05 was considered statistically significant, indicating a meaningful association or difference, while a ***p***-**value < 0.01** was considered highly important, highlighting particularly strong associations or differences.

## Results

### Mean nutrition literacy scores by gender and age

Table 1 shows the mean nutrition literacy by gender and age. The findings indicate that nutrition literacy significantly increases with age, with TNL and its domains improving across adolescence stages (*p* < 0.001). This progression is particularly evident in Functional Nutrition Literacy (FNL), where scores rise from 18.6 in early adolescence to 23.0 in late adolescence (*p* < 0.001), suggesting a developmental enhancement in fundamental nutrition knowledge. Gender differences were observed in specific nutrition literacy domains, with females scoring significantly higher in Interactive Nutrition Literacy (INL) and Critical Nutrition Literacy (CNL) compared to males (*p* = 0.001, *p* < 0.001, respectively). However, no significant gender differences were found in Functional Nutrition Literacy (FNL) (*p* = 0.814), indicating that both genders exhibit similar levels of basic nutrition comprehension. Overall, females demonstrated a higher TNL score than males (73.3 vs. 70.2, *p* < 0.001), reinforcing the trend of greater engagement with nutrition-related discussions and critical evaluation of dietary information among adolescent females.

**Table 1:** Nutrition literacy across adolescence stages and gender

Variables	FNL^(a)^(score: 7–35) (*n* = 1050) (mean ± SD)	INL^(b)^(score: 6–30)(*n* = 1050) (mean ± SD)	CNL^(c)^(score: 9–45)(*n* = 1050) (mean ± SD)	TNL^(d)^(score: 22–110) (*n* = 1050) (mean ± SD)
**Adolescence stage** ^**€**^				
• Early • Middle • Late *P value* ^**§**^	18.6 ± 6.7 19.8 ± 8.0 23.0 ± 6.5 < 0.001**	20.2 ± 5.5 20.1 ± 6.4 21.5 ± 5.1 0.002**	30.0 ± 3.7 29.8 ± 4.6 32.9 ± 5.7 < 0.001**	68.8 ± 7.0 69.7 ± 6.8 77.4 ± 11.7 < 0.001**
**Adolescence gender**				
• Female • Male *P value* **#**	20.5 ± 7.3 20.4 ± 7.3 0.814	21.1 ± 5.6 19.9 ± 5.8 0.001**	31.7 ± 4.8 29.9 ± 4.9 < 0.001**	73.3 ± 10.0 70.2 ± 8.8 < 0.001**

Tests of significance were: # t-test between two means, § ANOVA test between means, **highly significant < 0.01, (a) Functional Nutrition Literacy, (b) Interactive Nutrition Literacy, (c) Critical Nutrition Literacy, and (d) Total Nutrition Literacy, ^€^Early adolescence (10–13 years old), middle adolescence (14–16 years old), late adolescence (17–19 years old)

### Distribution of nutrition literacy levels

**Fig. 1:**
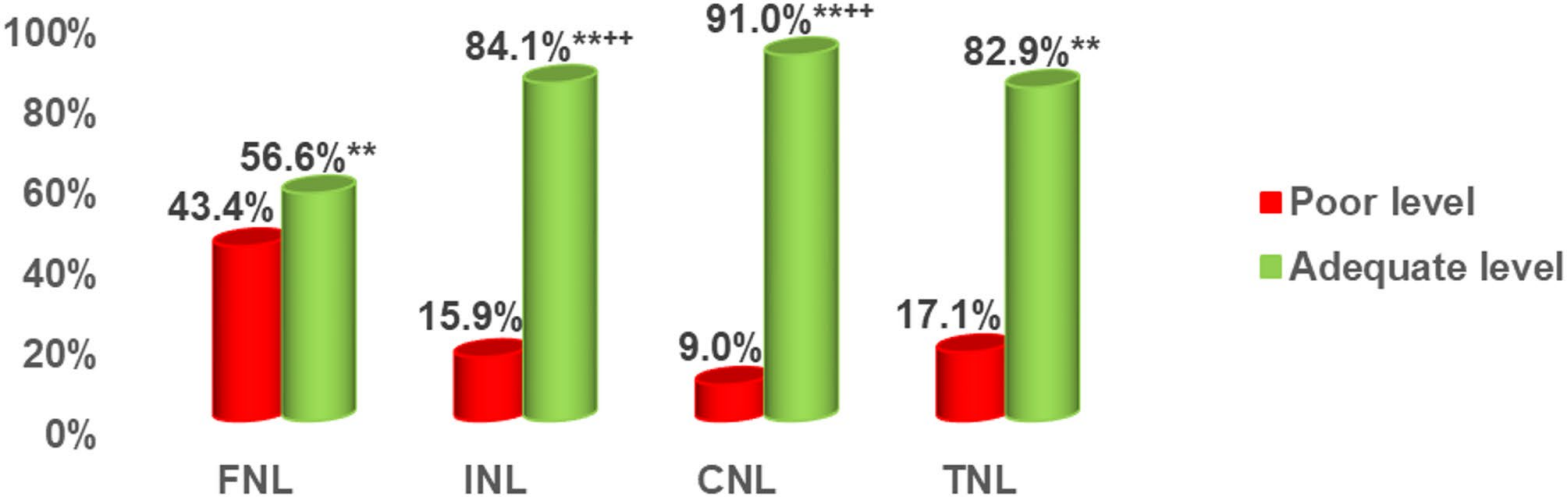
Percent distribution of adolescents’ nutrition literacy and its domains. Test of significance was: z test between two proportions, **highly significant < 0.01 between poor level and adequate level of each score, ^++^highly significant < 0.01 between adequate FNL and adequate INL as well as between adequate FNL and adequate CNL

Figure 1 displays the percent distribution of adolescents’ nutrition literacy level and its domains, across all adolescence stages. The proportion of adolescents with adequate Total Nutrition Literacy (TNL) was 82.9%, and adequacy levels for its domains; FNL, INL, and CNL levels were significantly higher than the inadequacy levels. Among these, the adequacy of FNL was notably lower at 56.6%, compared to higher adequacy levels for INL (84.1%) and CNL (91.0%) (*p* < 0.01).

**Fig. 2:**
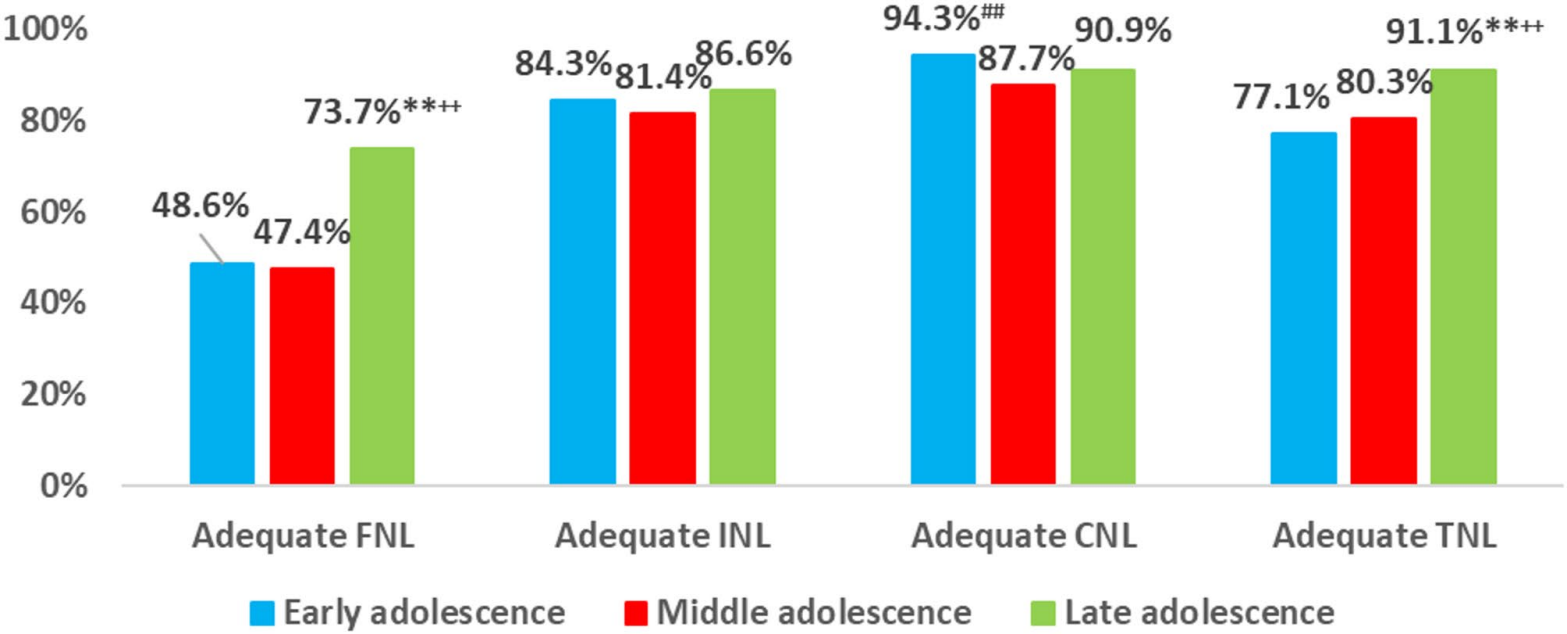
Percent distribution of adequate adolescents’ nutrition literacy according to the adolescence stage. Test of significant was: z test between two proportions, **highly significant < 0.01 between late adolescence and early adolescence, ^++^highly significant < 0.01 between late adolescence and middle adolescence, ^##^highly significant < 0.01 between early adolescence and middle adolescence

**In early adolescence**, adequate Critical Nutrition Literacy (CNL) levels were significantly higher at 94.3%, while adequate Functional Nutrition Literacy (FNL) remained lower at 48.6% (*p* < 0.01). **In middle adolescence**, adequate CNL decreased to 87.7%, with FNL still low at 47.4% (*p* < 0.01). **By late adolescence**, adequate Total Nutrition Literacy (TNL) rose significantly to 91.1%, and FNL adequacy also improved compared to earlier stages (Fig. 2).

### Nutrition literacy adequacy by adolescent stage (quartile-based analysis)

Table 2 presents the distribution of nutrition literacy adequacy across different literacy domains and adolescence stages, using median values and quartile analysis. Across all adolescence stages, the median scores for TNL and its domains—FNL, INL, and CNL—tend to cluster near the lower threshold of adequacy, indicating that while some adolescents attain sufficient literacy levels, the majority remain closer to the minimum adequacy range.

The findings highlight that **early adolescents** exhibit the lowest nutrition literacy scores, with 85.2% falling within the lowest quartile for TNL (range: 66–77). Similarly, 67.6% of early adolescents score in the lowest quartile for FNL, suggesting limited foundational knowledge of nutrition concepts. As adolescents transition into **middle adolescence**, gradual improvements emerge, particularly in FNL, where the median score increases from 21.5 to 25.0, although nearly half (49.4%) still remain in the lowest quartile. A notable shift is observed in INL, with 43.5% of middle adolescents progressing into the third quartile, suggesting increased engagement in nutrition-related discussions and decision-making.

The most significant improvements occur in late adolescence, where TNL adequacy diversifies across quartiles, with 37% in the second quartile and 12% in the highest quartiles, demonstrating a broader distribution of literacy competence. Moreover, CNL reaches a higher distribution in the upper quartiles (6.0%) among late adolescents, compared to 0% in early and middle adolescence, indicating a stronger ability to critically evaluate and apply nutrition information as adolescents mature.

**Table 2:** Distribution of nutrition literacy adequacy by literacy domain and adolescence stage (Median and Quartiles)

Variables	Overall	Adolescence stage with adequate TNL^@^(n = 870)			
		Early adolescence (10-<14 years old)	Middle adolescence (14-<17 years old)	Late adolescence (17–19 years old)	*P* value#
**Adequate level of FNL** ^(a)^ *[range: 21–35, median (IQR#)]*	**(n = 594)**	**(n = 170)**	**(n = 166)**	**(n = 258)**	< 0.001**
• 1st quartile adequacy	24.0 (21.0–28.0)	21.5 (21.0–25.0)	25.0 (21.0–35.0)	25.0 (22.0–28.0)	
*[range: 21–24, N (%)]*					
• 2nd quartile adequacy	313 (52.7)	115 (67.6)	82 (49.4)	116 (45.0)	
*[range: 25–28, N (%)]*					
• 3rd quartile adequacy	134 (22.6)	25 (14.7)	29 (17.5)	80 (31.0)	
*[range: 29–32, N (%)]*					
• 4th quartile adequacy	47 (7.9)	10 (5.9)	6 (3.6)	31 (12.0)	
*[range: 33–35, N (%)]*					
	100 (16.8)	20 (11.8)	49 (29.5)	31 (12.0)	
**Adequate level of INL** ^(b)^ *[range: 18–30, median (IQR* ^*#*^ *)]*	**(n = 883)**	**(n = 295)**	**(n = 285)**	**(n = 303)**	< 0.001**
• 1st quartile adequacy	23.0 (19.0–24.0)	22.0 (18.0–24.0)	24.0 (19.0–24.0)	24.0 (21.0–24.0)	
*[range: 18–20, N (%)]*					
• 2nd quartile adequacy	302 (34.2)	140 (47.5)	94 (33.0)	68 (22.4)	
*[range: 21–23, N (%)]*					
• 3rd quartile adequacy	140 (15.9)	35 (11.9)	40 (14.0)	65 (21.5)	
*[range: 24–26, N (%)]*					
• 4th quartile adequacy	353 (40.0)	90 (30.5)	124 (43.5)	139 (45.9)	
*(range: 27–30, N (%)]*					
	88 (10.0)	30 (10.2)	27 (9.5)	31 (10.2)	
**Adequate level of CNL** ^(c)^ *[range: 27–45, median (IQR* ^*#*^ *)]*	**(n = 955)**	**(n = 330)**	**(n = 307)**	**(n = 318)**	< 0.001**
• 1st quartile adequacy	31.0 (29.0–34.0)	30.0 (27.0–33.0)	30.0 (30.0–33.0)	34.0 (30.0–36.0)	
*[range: 27–31, N (%)]*					
• 2nd quartile adequacy	513 (53.7)	225 (68.2)	193 (62.9)	95 (29.9)	
*[range: 32–36, N (%)]*					
• 3rd quartile adequacy	334 (35.0)	95 (28.8)	90 (29.3)	149 (46.9)	
*[range: 37–41, N (%)]*					
• 4th quartile adequacy	89 (9.3)	10 (3.0)	24 (7.8)	55 (17.3)	
*[range: 42–45, N (%)]*					
	19 (2.0)	0 (0.0)	0 (0.0)	19 (6.0)	
**Adequate level of TNL** ^(d)^ *[range: 66–110, median (IQR* ^*#*^ *)]*	**(n = 870)**	**(n = 270)**	**(n = 281)**	**(n = 319)**	< 0.001**
• 1st quartile adequacy	70.0 (68.0–80.0)	68.0 (66.0–72.0)	68.0 (67.0–76.0)	78.0 (70.0–86.0)	
*[range: 66–77, N (%)]*					
• 2nd quartile adequacy	614 (70.6)	230 (85.2)	234 (83.3)	150 (47.0)	
*[range: 78–88, N (%)]*					
• 3rd quartile adequacy	194 (22.3)	35 (13.0)	41 (14.6)	118 (37.0)	
*[range: 89–99, N (%)]*					
• 4th quartile adequacy	47 (5.4)	5 (1.9)	6 (2.1)	36 (11.3)	
*[range: 100–110, N (%)]*					
	15 (1.7)	0 (0.0)	0 (0.0)	15 (4.7)	

# test of significance was: X^2^ between the groups, **highly significant < 0.01, (a) Functional Nutrition Literacy, (b) Interactive Nutrition Literacy, (c) Critical Nutrition Literacy, and (d) Total Nutrition Literacy, ^*#*^IQR: interquartile range,

### Gender differences in nutrition literacy

**Fig. 3:**
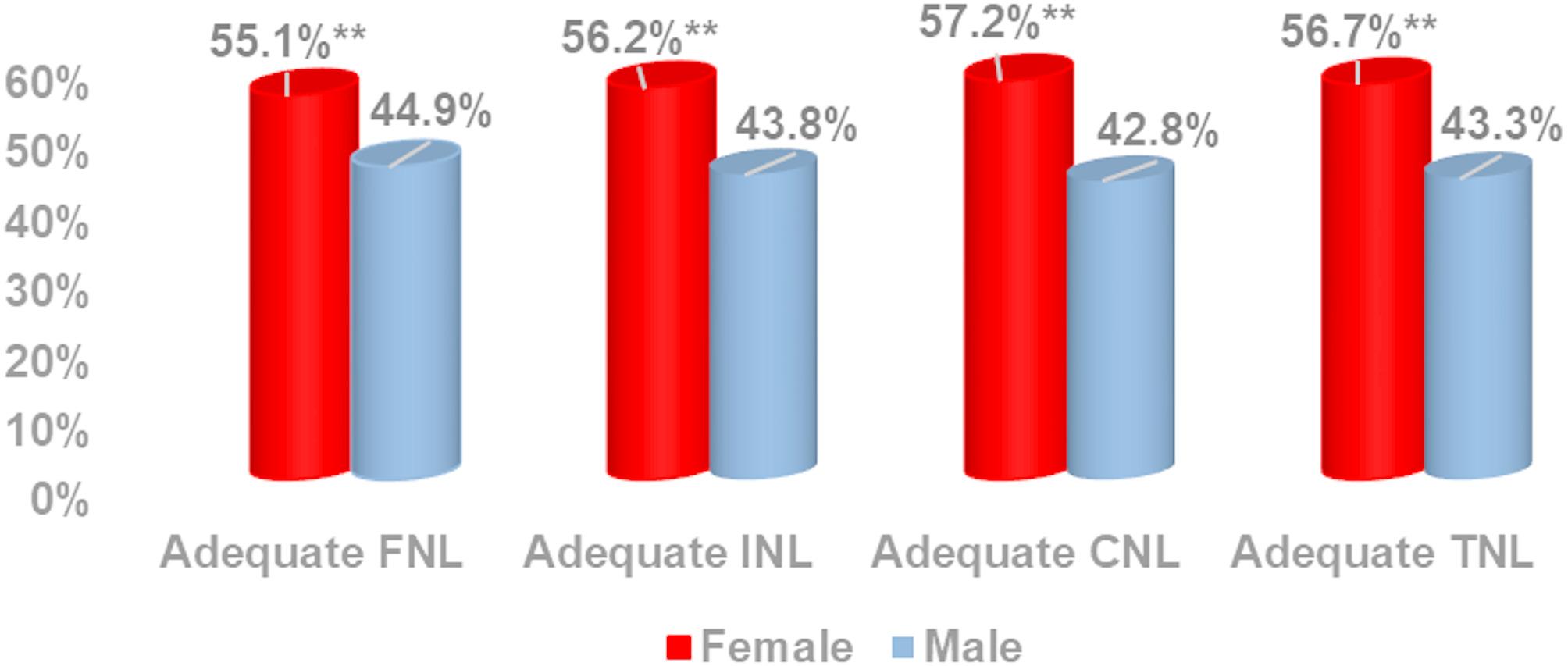
Percent distribution of adequate adolescents’ nutrition literacy among the studied participants according to the adolescent gender. Test of significance was: z test between two proportions, **significant < 0.01 between females and males, FNL: functional nutrition literacy, INL: interactive nutrition literacy, CNL: critical nutrition literacy, TNL: total nutrition literacy

Adequate levels of Total Nutrition Literacy (TNL) and its domains—Functional (FNL), Interactive (INL), and Critical Nutrition Literacy (CNL)—were significantly higher among females than males (*p* < 0.01) (Fig. 3).

### Influences of various adolescent and parental factors on total nutrition literacy scores

Table 3 presents the influence of various adolescent and parental factors on Total Nutrition Literacy (TNL) scores across the three adolescence stages: early (10–13 years), middle (14–16 years), and late adolescence (17–19 years).

The findings reveal a complex interplay between **BMI**,** parental factors**,** household income**, and TNL across adolescence stages. In early adolescence, underweight adolescents exhibit significantly higher TNL scores than their normal-weight and overweight/obese peers (*p* < 0.001), suggesting that health concerns or parental influence may contribute to better nutrition awareness. However, in middle adolescence, overweight/obese adolescents score significantly higher in TNL (*p* < 0.001) than their normal-weight and underweight counterparts, possibly reflecting increased awareness of nutrition due to concerns about weight management. By late adolescence, the association between BMI and TNL becomes non-significant, indicating that factors beyond weight status—such as personal health interest, education, and environmental influences—play a more dominant role in shaping nutrition literacy at this stage.

#### Parental education

emerges as a strong determinant of adolescent TNL, with higher parental education (both mother and father) significantly associated with better TNL scores across all adolescence stages (*p* < 0.001). This reinforces the role of parental education in shaping adolescent nutrition knowledge, as adolescents with highly educated parents consistently demonstrate stronger nutrition literacy skills. Similarly, **parental food literacy (FL)** is a critical factor, as adolescents whose parents have adequate FL score significantly higher in TNL (*p* < 0.001 in early adolescence). This suggests that parental nutrition knowledge directly influences adolescents’ ability to understand and apply dietary information, emphasizing the importance of family-based nutrition education. Meanwhile, **Household income** is inversely associated with TNL, as adolescents from lower-income households (< 5000 EP) demonstrate significantly higher TNL scores across all adolescence stages (*p* < 0.001).

Larger family size also positively impacts TNL, with adolescents from bigger households scoring higher (*p* < 0.001), perhaps due to structured meal routines and shared responsibilities, and adolescents with parents who have chronic diseases score slightly higher in TNL (*p* < 0.001), likely due to health-related discussions in these families.

**Table 3:** Effect of adolescent and parental factors on total nutrition literacy (TNL) scores across adolescence stages

Variables	Studied Adolescents (n = 1000)		
	TNL^(a)^ (score: 22–110)		
	Early adolescence (10–13 years old) (n = 350)	Middle adolescence (14–16 years old) (n = 350)	Late adolescence (17–19 years old) (n = 350)
	**(mean ± SD)**	**(mean ± SD)**	**(mean ± SD)**
**Adolescent Factors**			
**Adolescent gender**			
Females	69.0 ± 8.0	69.4 ± 5.5	80.6 ± 10.4
Males	68.5 ± 5.6	70.1 ± 7.9	72.4 ± 11.9
*P value#*	0.547	0.318	< 0.001**
**Adolescent weight status (BMI)**			
Normal-weight	67.8 ± 6.1	69.0 ± 5.1	77.4 ± 11.5
Underweight	73.5 ± 10.5	69.5 ± 12.1	80.9 ± 12.8
Overweight / obese	69.8 ± 6.4	75.6 ± 11.7	76.8 ± 11.8
*P value*§	< 0.001**	< 0.001**	0.348
**Parental Factors**			
**Parent age group**			
< 40 years	69.5 ± 8.1	74.9 ± 11.8	79.2 ± 8.0
≥ 40 years	68.3 ± 6.1	69.1 ± 5.7	77.1 ± 12.1
*P value#*	0.127	< 0.001**	0.14
**Mothers’ education level**			
School level	68.2 ± 6.8	69.5 ± 5.8	74.0 ± 11.9
University level or higher	71.7 ± 7.5	70.7 ± 10.6	80.4 ± 10.5
*P value#*	0.001**	0.426	< 0.001**
**Fathers’ education level**			
School level	67.4 ± 2.8	69.4 ± 11.0	74.6 ± 12.1
University level or higher	68.9 ± 7.3	69.8 ± 5.7	80.8 ± 10.4
*P value#*	0.020*	0.785	< 0.001**
**Primary caregiver**			
Both parents	68.6 ± 7.2	70.0 ± 7.1	77.9 ± 12.0
Either parent (mother or father)	70.0 ± 5.3	68.5 ± 5.3	74.4 ± 8.9
*P value#*	0.277	0.102	0.016*
**Parent marital status**			
Married	68.9 ± 7.2	70.3 ± 7.0	77.6 ± 11.3
Not married	67.0 ± 1.0	65.7 ± 2.9	76.6 ± 13.3
*P value#*	< 0.001**	< 0.001**	0.542
**Number of children**			
≤ 3 children	68.7 ± 6.9	66.3 ± 3.4	76.3 ± 8.5
> 3 children	70.0 ± 8.9	69.9 ± 6.8	83.6 ± 11.2
*P value#*	0.592	0.001**	< 0.001**
**Parent employment status**			
Not employed	68.4 ± 6.2	69.6 ± 6.7	77.3 ± 11.8
Employed	75.5 ± 13.7	72.7 ± 6.6	80.4 ± 8.6
*P value#*	0.032*	0.066	0.288
**Parent monthly income**			
Less than 5000 EP	71.7 ± 9.1	70.0 ± 7.1	80.9 ± 8.9
More than 5000 EP	67.3 ± 5.2	67.6 ± 2.8	75.9 ± 12.4
*P value#*	< 0.001**	< 0.001**	< 0.001**
**Household crowding index**			
Not crowded (≤ 1)	67.6 ± 5.0	67.1 ± 4.0	74.4 ± 9.4
Crowded (> 1)	70.2 ± 8.6	73.4 ± 8.1	79.6 ± 12.3
*P value#*	0.001**	< 0.001**	< 0.001**
**Parent weight status (BMI)**			
Normal-weight	68.4 ± 7.6	62.0 ± 0.0	78.9 ± 11.9
Underweight	---	69.1 ± 5.4	75.7 ± 3.7
Overweight / obese	69.0 ± 6.7	70.2 ± 7.3	77.0 ± 11.7
*P value*§	0.422	0.014*	0.441
**Parent had one or more chronic disease**			
No	70.0 ± 8.9	70.1 ± 7.0	80.1 ± 10.3
Yes	67.9 ± 5.3	67.6 ± 5.3	75.4 ± 12.2
*P value#*	0.013*	0.009**	< 0.001**
**Parental total FL** ^**(b)**^			
Poor	66.4 ± 3.3	69.2 ± 6.0	76.7 ± 11.9
Adequate	69.8 ± 7.9	70.2 ± 7.4	77.8 ± 11.6
*P value#*	< 0.001**	0.145	0.432

Tests of significance were: ^#^t-test between two means, and § ANOVA test between means, (a) TNL: Total Nutrition Literacy, (b) FL: Food Literacy, *significant < 0.05, **highly significant < 0.01

### Predictors of adequate total nutrition literacy

Table 4 provides a logistic regression analysis identifying predictors of TNL for adolescents at different stages (early, middle, and late adolescence). The analysis includes both adolescent factors (gender, weight status) and parental factors (age, education, marital status, caregiver type, employment, income, crowding, BMI, chronic disease status, and food literacy). The findings from Table 4 indicate that parental factors play a significant role in shaping adolescent Total Nutrition Literacy (TNL), particularly in early and middle adolescence.

#### In early adolescence (10–13 years)

adolescents with employed parents are significantly more likely to achieve adequate TNL (AOR = 3.9, 95%CI: 1.5–10.5, *p* < 0.01), suggesting that economic stability and resources may play a key role in supporting early nutrition literacy. Additionally, parental food literacy was found to be a crucial factor, as adolescents with parents who had inadequate food literacy were significantly less likely to achieve adequate TNL (AOR = 2.5, 95%CI: 1.0–6.0, *p* < 0.05), emphasizing the importance of parents’ nutritional knowledge in shaping young adolescents’ understanding of of dietary choices and healthy eating habits.

#### In middle adolescence (14–16 years)

The absence of parental chronic disease is significantly associated with lower adolescent TNL (AOR = 0.3, 95%CI: 0.1–0.7, *p* < 0.05). Parental FL also remains a significant predictor of TNL at this stage (AOR = 1.9, 95%CI: 1.0–3.7, *p* < 0.05). **In late adolescence (17–19 years old)**, no significant predictors of TNL were identified.

**Table 4:** Multiple logistic regression analysis for predictors of adequate total nutrition literacy (TNL) by adolescence stage

Parameters#	Early adolescence		Middle adolescence		Late adolescence	
	(10–13 years old)		(14–16 years old)		(17–19 years old)	
	AOR	CI	AOR	CI	AOR	CI
**Adolescent factors**						
**Adolescence gender** (female is the base)	1.2	0.7	1.2	0.6	1.3	0.5
		2.2		2.3		3.5
**Adolescence weight status (BMI)** (normal-weight vs. underweight)	0.5	0.2	1	0.2	0.7	0.1
		1.3		5.6		3.3
**Adolescence weight status (BMI)** (normal-weight vs. overweight/ obese)	0.9	0.4	1.2	0.6	1	0.3
		2		2.3		3.7
**Parent factors**						
**Parent age group** (< 40 years is the base)	1	0.6	2.5	0.6	1	0.3
		1.9		10.3		3.2
**Mother education level** (university level is the base)	1.4	0.6	1.6	0.5	2.4	0.5
		3.7		5.6		11.4
**Father education level** (university level is the base)	2.7	0.7	1.1	0.4	1.2	0.5
		10.1		3.4		5.8
**Primary caregiver**(both parents is the base)	2.5	0.7	0.9	0.3	1.4	0.4
		8.7		3.1		5
**Parent marital status** (married is the base)	1.1	0.3	2.5	0.6	1.4	0.3
		4.6		10.1		5.4
**Number of children in the household** (> 3 children is the base)	0.9	0.3	1.2	0.4	1.7	0.3
		2.6		4.2		9
**Parent employment status** (employed is the base)	3.9**	1.5	2.1	0.5	0.9	0.1
		10.5		9.1		12
**Monthly income** (more than 5000 EP is the base)	1	0.4	0.8	0.5	0.6	0.1
		2.2		2.5		2.8
**Household crowding index**(crowded [> 1] is the base)	1	0.4	0.9	0.5	1.2	0.4
		2.8		1.8		3.8
**Parent weight status (BMI)** (normal-weight vs. underweight)	-----	-----	0.7	0.1	1.1	0.1
		-----		4.9		10.8
**Parent weight status (BMI)** (normal-weight vs. overweight/ obese)	0.6	0.3	0.5	0.2	2.2	0.7
		1.2		1.1		6.6
**Parent had one or more chronic disease** (no is the base)	1.1	0.4	0.3*	0.1	2.1	0.5
		3.4		0.7		9.9
**Parental total FL**^**(a)**^(adequate is the base)	2.5*	1	1.9*	1	1.6	0.6
		6		3.7		4.8
Model fit (Cox & Snell R Square)	0.096		0.103		0.061	
Percent correctly predicted (Hosmer and Leme show Test)	0		0		0.844	

^#^Analyzed using enter multivariate logistic regression, AOR: Adjusted Odds Ratio, CI: Confidence Interval, *significant < 0.05, **highly significant < 0.01. Variable(s) entered in each model: adolescents (gender and adolescent BMI) and parents (age, education level, marital status, number of children, primary caregiver, job status, monthly income, household crowding index, had one or more chronic disease, vitamins intake, minerals intake, and (a) parental total food literacy)

### Associations between nutrition literacy and nutritional outcomes

Table 5 examined how different aspects of adolescents’ nutrition literacy—FNL, INL, CNL, and TNL—are associated with various nutritional outcomes, including BMI categories (underweight, overweight/obese, and normal weight), vitamin intake, and mineral intake. The findings suggest distinct relationships between nutrition literacy domains and BMI, vitamin intake, and mineral intake among adolescents.

For **BMI**, adequate **FNL** is linked to a significantly higher likelihood of adolescents being underweight (COR = 0.4, 95%CI: 0.2–0.7, *p* < 0.01), though it has no significant effect on overweight or obesity. **INL** has the opposite effect, as adolescents with adequate **INL** are less likely to be underweight (COR = 4.0, 95%CI: 2.4–6.6, *p* < 0.01) but 40% more likely to be overweight/obese (COR = 0.4, 95%CI: 0.2–0.7, *p* < 0.01). **CNL** does not significantly affect underweight status, but it does correlate with a 30% higher likelihood of overweight or obesity (COR = 0.3, 95%CI: 0.1–0.6, *p* < 0.01). In contrast, TNL does not show a significant effect on BMI categories.

When it comes **to vitamin intake**, adequate FNL increases the likelihood of intake (COR = 1.6, 95%CI: 1.3–2.1, *p* < 0.01), while adequate INL is associated with twice the likelihood of vitamin intake (COR = 2.0, 95%CI: 1.3–2.9, *p* < 0.01). CNL has the most substantial effect on vitamin intake, with a COR of 3.6 (95%CI: 2.0–6.6, *p* < 0.01). Adolescents with adequate TNL are nearly five times more likely to report vitamin intake (COR = 4.9, 95%CI: 3.1–7.9, *p* < 0.01), highlighting the comprehensive impact of total nutrition literacy on vitamin consumption.

**For mineral intake**, adequate FNL is associated with a greater likelihood of intake (COR = 2.4, 95%CI: 1.8–3.3, *p* < 0.01), while adequate INL doubles the likelihood of mineral intake (COR = 2.0, 95%CI: 1.3–3.1, *p* < 0.01). CNL also significantly increases the likelihood of mineral intake three times (COR = 3.8, 95%CI: 1.9–7.7, *p* < 0.01), and TNL has the strongest association, increasing the likelihood of mineral intake nearly six-fold (COR = 5.8, 95%CI: 3.2–10.3, *p* < 0.01). These results emphasize the role of nutrition literacy, particularly total and critical literacy, in promoting better dietary supplement practices among adolescents.

**Table 5:** Association between adolescents’ nutrition literacy levels and health nutritional outcome: BMI, vitamin intake, and mineral intake

Variables§	Studied adolescents (n = 1050)						
	BMI			Vitamins intake®		Minerals intake®	
	Underweight	Overweight/ Obese	Normal weight	No	Yes	No	Yes
	**n = 70**	**n = 206**	**n = 774**	**n = 687**	**n = 363**	**n = 768**	**n = 282**
	**N (%)**	**N (%)**	**N (%)**	**N (%)**	**N (%)**	**N (%)**	**N (%)**
**Adolescents FNL** ^**(a)**^							
Poor (n = 456)							
Adequate (n = 594)	16 (22.9)	98 (47.6)	342 (44.2)	327 (47.6)	129 (35.5)	376 (49.0)	80 (28.4)
	54 (77.1)	108 (52.4)	432 (55.8)	360 (52.4)	234 (64.5)	392 (51.0)	202 (71.6)
**COR (95% CI)#**	0.4 (0.2–0.7)**	1.1 (0.8–1.6)		1.6 (1.3–2.1)**		2.4 (1.8–3.3)**	
**Adolescents INL** ^**(b)**^							
Poor (n = 167)							
Adequate (n = 883)	30 (42.9)	14 (6.8)	123 (15.9)	129 (18.8)	38 (10.5)	139 (18.1)	28 (9.9)
	40 (57.1)	192 (93.2)	651 (84.1)	558 (81.2)	325 (89.5)	629 (81.9)	254 (90.1)
**COR (95% CI)#**	4.0 (2.4–6.6)**	0.4 (0.2–0.7)**		2.0 (1.3–2.9)**		2.0 (1.3–3.1)**	
**Adolescents CNL** ^**(c)**^							
Poor (n = 95)							
Adequate (n = 955)	8 (11.4)	6 (2.9)	81 (10.5)	82 (11.9)	13 (3.6)	86 (11.2)	9 (3.2)
	62 (88.6)	200 (97.1)	693 (89.5)	605 (88.1)	350 (96.4)	682 (88.8)	273 (96.8)
**COR (95% CI)**	1.1 (0.5–2.4)	0.3 (0.1–0.6)**		3.6 (2.0-6.6)**		3.8 (1.9–7.7)**	
**Adolescents TNL** ^**(d)**^							
Poor (n = 180)	16 (22.9)	36 (17.5)	128 (16.5)	159 (23.1)	21 (5.8)	167 (21.7)	13 (4.6)
Adequate (n = 870)	54 (77.1)	170 (82.5)	646 (83.5)	528 (76.9)	342 (94.2)	601 (78.3)	269 (95.4)
**COR (95% CI)#**	1.5 (0.8–2.7)	1.1 (0.7–1.6)		4.9 (3.1–7.9)**		5.8 (3.2–10.3)**	

®“Vitamin intake” and “Mineral intake” refer to self-reported use of dietary supplements, not total intake or adequacy relative to dietary reference values.(a) Functional Nutrition Literacy, (b) Interactive Nutrition Literacy, (c) Critical Nutrition Literacy, and (d) Total Nutrition Literacy, #test of significance was: X^2^ between the groups, **highly significant < 0.01, COR: Crude odds Ratio, § the cut off score of ≥ 21 indicating adequate FNL, the cut off score of ≥ 18 indicating adequate INL, the cut off score of ≥ 27 indicative of CNL and cut off score of ≥ 66 reflected adequate TNL

## Discussion

Understanding nutrition literacy development across adolescence provides valuable insights for designing age-appropriate health education strategies. This study examined over 1,500 adolescents aged 10 to 19, using both descriptive and analytical approaches to assess functional (FNL), interactive (INL), and critical (CNL) nutrition literacy across early, middle, and late adolescence. The findings highlight key determinants, including age, gender, parental education, and family background, offering a comprehensive perspective on nutrition literacy progression in Egypt.

### Age and gender-related differences in nutrition literacy

Nutrition literacy improves significantly with age, with late adolescents (17–19 years) demonstrating the highest TNL scores, particularly in interactive and critical domains. This suggests that cognitive maturity and increased exposure to health information enhance nutrition knowledge and engagement [72]. Similar findings were reported in a Saudi study, where younger adolescents had a significantly higher prevalence of inadequate nutrition literacy (53.9%) compared to middle-aged (48.2%) and older adolescents (41.9%) (*p* < 0.001) [73]. However, functional literacy remained low across all stages, emphasizing the need for reinforcing foundational education.

Female adolescents consistently outperformed males in nutrition literacy, particularly in interactive literacy, indicating greater engagement with nutrition-related information in social settings. This aligns with Bookari’s research in Saudi Arabia, which found that male gender doubled the risk of poor nutrition literacy [72]. However, functional literacy scores were comparable between genders (*p* > 0.05), suggesting similar foundational nutrition knowledge acquisition. These findings underscore the importance of age-specific and gender-sensitive interventions to improve nutrition literacy at different adolescence stages.

### Quartile distribution of nutrition literacy adequacy

Unlike traditional studies that classify nutrition literacy (NL) as adequate or inadequate, this study utilized quartile distribution to offer a more detailed and comprehensive assessment of literacy levels across adolescence. This approach allows for a better understanding of literacy disparities by identifying varying levels of proficiency and highlighting subgroups that require targeted interventions.

**In early adolescence (10–13 years**), 85.2% of adolescents scored in the lowest quartile for TNL, with 67.6% falling in the lowest quartile for FNL (median = 21.5). This suggests that fundamental nutrition knowledge is weak during this stage. Moreover, INL remained low (median = 22.0), indicating limited engagement with nutrition-related discussions. Similarly, CNL scores were among the lowest (median = 30.0), reflecting an inability to critically assess or apply nutrition information. These findings highlight the urgent need for targeted interventions focusing on foundational nutrition education, particularly emphasizing basic nutrition knowledge and interactive learning methods to build early engagement with dietary information. Research suggests that nutrition education efforts in younger adolescents should prioritize interactive learning strategies to improve basic understanding and application of nutrition knowledge [74].

As adolescents transition into **middle adolescence (14–16 years**), moderate improvements in nutrition literacy begin to emerge. The most notable change was observed in INL (3rd quartile = 43.5%), suggesting that adolescents at this stage are more likely to engage with and discuss nutrition-related topics. However, CNL remained weak, indicating that while middle adolescents are becoming more interactive in nutrition discussions, they still struggle with evaluating and applying nutritional information critically. Previous studies have indicated that this stage marks a shift toward increased autonomy in food choices, making nutrition literacy interventions particularly important in helping adolescents navigate misleading food marketing and dietary misinformation [75].

By late adolescence (17–19 years), a significant upward shift in literacy distribution is observed, with higher quartile representation across all domains. This stage is characterized by improved foundational knowledge, greater engagement in nutrition discussions, and stronger critical thinking skills related to nutrition. Late adolescents demonstrate a greater ability to assess, interpret, and apply nutrition information, making this stage particularly critical for reinforcing long-term healthy behaviors. Studies suggest that cognitive development during this phase allows for more advanced reasoning and decision-making regarding health and nutrition choices [28].

### Influence of adolescent and parental factors on total nutrition literacy (TNL) across stages

**This study emphasizes** the dynamic influences of adolescent and parental factors on TNL across adolescence. **Early adolescents** show significant influences from parental education, and employment suggesting a foundational reliance on parental support for nutrition knowledge. **Middle adolescents** appear more affected by family structure, such as marital status and family size, indicating a growing influence of social context. In **late adolescence**, TNL is shaped more by individual factors continues to benefit from parental education. This finding indicates that parental support remains crucial, as evidenced by higher TNL scores among adolescents with dual caregivers and higher parental education. At the same time, it reflects the transition toward more independent and socially influenced nutrition literacy.

These findings align with Hoteit et al. [47], who reported that adolescents with parents possessing high food literacy had greater odds of achieving high TNL. Similarly, Bookari’s study [73] reinforced the idea that parental influence remains a major factor in shaping adolescent dietary behaviors. Research also confirms strong correlations between parental nutrition literacy, household dietary quality, and adolescent dietary habits [76–79]. Research postulated that parents influence their children’s eating behaviors and food choices through their own dietary practices and feeding techniques, serving as role models and actively making meal choices for the family [80, 81]. These insights support the need for tailored nutrition education interventions that integrate parental involvement at early stages while fostering adolescent independence in later stages.

### Predictors of adequate total nutrition literacy (TNL) at different adolescence stages

Findings reveal that parental employment and food literacy strongly influence TNL in early adolescence. Parental education positively correlates with healthier dietary habits, while lower parental education is associated with higher fizzy drink intake [82, 83].

**In middle adolescence**,** t**he absence of parental chronic disease is significantly associated with lower adolescent TNL, indicating that the non healthier family environment might encourage better nutrition practices in adolescents to stay healthy. Parental FL also remains a significant predictor of TNL at this stage highlighting the continued influence of parents’ nutritional knowledge on adolescents’ understanding of healthy eating during this transitional phase. Hoteit et al. found that adolescents with chronically ill parents were often nutritionally illiterate [84]. As adolescents move into **late adolescence**, no significant predictors of TNL were identified. This suggests that external factors, such as self-driven learning, peer influence, or independent access to nutrition information, become more dominant in shaping nutrition literacy at this stage [85]. Unlike early and middle adolescence, where parental factors play a substantial role, late adolescents may rely more on personal experiences and external resources rather than direct parental influence.

### Associations between nutrition literacy domains and nutritional outcomes

All nutrition literacy domains (FNL, INL, and CNL) significantly influence vitamin and mineral intake, with TNL and CNL showing the strongest associations. This suggests that both general nutrition knowledge and critical thinking skills play vital roles in encouraging healthy dietary supplementation [51– [63, 86].

The relationship between nutrition literacy and BMI is more complex: Higher FNL is associated with a greater likelihood of being underweight, possibly due to increased awareness of calorie intake and restrictive dietary habits. INL and CNL exhibit mixed relationships with overweight and obesity, indicating that nutrition literacy alone may not directly influence weight status. These findings align with a Chinese study [87], which found an inverse association between nutrition literacy and overweight/obesity in senior high school students. Conversely, other studies have shown that students with lower-educated parents are more likely to be overweight or obese, supporting findings in existing literature [82, 83]. However, Taleb and Itani [88] found no significant correlation between nutrition literacy and BMI, highlighting the need for further research on this relationship.

### Inverse association between household income and nutrition literacy

Surprisingly, household income is inversely associated with TNL, as adolescents from lower-income households (< 5000 EP) demonstrate significantly higher TNL scores across all adolescence stages (*p* < 0.001). This unexpected trend may be attributed to greater involvement in household food planning and budgeting, higher exposure to government or school-based nutrition programs, and a stronger reliance on traditional home-cooked meals that emphasize nutrition awareness. These findings highlight that nutrition literacy is not solely dependent on financial resources but is also shaped by environmental, educational, and familial influences, suggesting that nutrition education programs should target both high- and low-income groups to ensure equitable improvements in adolescent nutrition literacy. The inverse association between household income and TNL contrasts with existing research that typically links higher socioeconomic status (SES) to better nutrition literacy and healthier dietary behaviors [89]. Similarly, other research indicated that lower-income adolescents tend to have lower-quality diets while consuming more added sugars [90]. However, our results are similar to other study suggesting that parental education and food literacy play a more influential role than income, as adolescents with higher parental education exhibit better nutrition literacy, regardless of economic status [47].

### Strengths

This study demonstrated a comprehensive approach to analyzing adolescent nutrition literacy by examining total nutrition literacy (TNL) and its functional, interactive, and critical domains. This multi-dimensional perspective captured the complexity of nutrition literacy, revealing how these skills develop across different age groups and genders. Additionally, the study’s large, diverse sample of 1,050 adolescents, selected from various geographical and socioeconomic backgrounds, ensured a robust representation of Egyptian youth, enhancing the generalizability of the findings to similar contexts. A key strength of this study was its use of quartile distribution to evaluate literacy adequacy. This approach offered a more nuanced understanding of literacy levels by categorizing them across a spectrum, rather than treating literacy as a simple adequate/inadequate measure. Quartile analysis identifies which segment of the population fall into the lowest quartiles, signaling a greater need for support. This approach allows researchers and educators to monitor shifts within quartiles over time and to assess incremental improvements in literacy, which is particularly valuable in evaluating the impact of educational interventions.

Being a comprehensive study within the Egyptian context, the study’s findings may have wide generalizability to regions with similar educational systems, cultural norms, and socioeconomic conditions.

A key strength of the study is its detailed examination of influencing factors, such as adolescent and parental characteristics, including age, gender, BMI, education, and health status. This allows for a nuanced understanding of how family dynamics and demographic variables shape adolescents’ nutrition knowledge. The practical relevance of these findings is significant, as they highlight the impact of parental food literacy, education, and family health status on adolescents’ nutrition literacy levels. Such insights are valuable for policymakers, educators, and healthcare providers to create targeted interventions aimed at improving adolescent nutrition literacy and health.

### Limitations

The cross-sectional design limited causal inferences between nutrition literacy and influencing factors. Reliance on self-reported data may introduce biases, and the study’s focus on family dynamics lacked the examination of peer influence—a key factor in adolescence. Additionally, measuring nutrition literacy through BMI and vitamin/mineral intake might not fully capture the range of dietary behaviors linked to literacy. Expanding outcome measures to include broader health indicators could provide a more comprehensive understanding of nutrition literacy’s impact on adolescent health.

Moreover, intake of vitamins and minerals in this study was based solely on reported use of dietary supplements and did not assess adequacy in terms of total dietary intake or nutritional recommendations. Also, the dietary assessment of vitamins and minerals from food sources was conducted in a separate component of the research that is currently under review and could not be cited or fully integrated here.

### Conclusions and recommendations

This study highlighted the critical role of nutrition literacy (NL) development across adolescence, particularly in the Egyptian context, where age, gender, and familial factors significantly shape adolescents’ understanding of nutrition. Findings indicate that TNL and its domains—FNL, INL, and CNL—improved with age, reaching their highest levels in late adolescence. Gender differences were evident, with females demonstrating greater proficiency, particularly in interactive and critical literacy. Parental factors, including education, food literacy, health status, and family structure, strongly influence adolescent NL, emphasizing the importance of a supportive family environment in shaping dietary habits. Additionally, adequate TNL correlates positively with healthier BMI status and increased vitamin and mineral intake. However, the consistently lower adequacy of FNL underscores the need for stronger foundational nutrition education during early adolescence.

The study’s findings reinforce the importance of early exposure to nutrition-related content, demonstrating that nutrition education introduced in early adolescence significantly impacts diet, nutrient intake, and overall nutritional status. Adolescents who receive nutrition education in primary school settings exhibit healthier dietary behaviors, improved nutrient intake, and better nutritional outcomes in later adolescence. Research suggests that early adolescence is a critical period for establishing lifelong dietary habits, as cognitive and behavioral development during this stage influences how adolescents engage with and apply nutrition knowledge [91–94]. School-based nutrition education programs have been shown to increase awareness of healthy eating, encourage balanced food choices, and reduce the risk of obesity and nutrient deficiencies [95–97]. By integrating nutrition education into early adolescence curricula, public health strategies can ensure that adolescents develop a strong foundation in nutrition literacy, fostering lifelong healthy eating behaviors.

Despite these insights, existing policies in Egypt do not adequately address adolescent nutritional needs, particularly in rural areas, where dietary disparities persist [98]. Unlike higher-income countries, Egypt lacks comprehensive, school-based NL programs, especially those that incorporate interactive and critical literacy components necessary for informed food choices [99]. Addressing these gaps requires NL programs tailored to cultural and socioeconomic contexts. Given that Egyptian adolescents’ dietary behaviors are influenced by tradition, socioeconomic status, and regional disparities, studies from Middle Eastern populations suggest that nutrition education aligned with local dietary customs fosters greater engagement and sustainable dietary improvements [100]. Evidence from various interventions indicates that programs utilizing **culturally relevant channels and materials** consistently achieve **higher engagement and comprehension**, regardless of the health topic [101–106]. Incorporating traditional Egyptian food examples can enhance both comprehension and the practical application of nutrition knowledge [107].

Based on these findings, several key recommendations emerge to support nutrition literacy development among adolescents. Early adolescence should focus on foundational nutrition literacy (FNL), ensuring a strong base for interactive and critical literacy skills to develop later. Gender-specific interventions are necessary, as males tend to engage less in nutrition-related discussions, requiring strategies to increase their participation. Family involvement is another key component, as parental food literacy significantly influences adolescent nutrition knowledge. Providing workshops and resources for parents can reinforce nutrition education at home, fostering a health-conscious environment.

At the policy level, integrating structured nutrition literacy curricula across educational stages is essential to ensure steady knowledge development from early to late adolescence. Further longitudinal studies should explore the long-term impact of nutrition literacy on adolescent health outcomes and identify effective strategies to address socioeconomic disparities. By implementing culturally relevant, stage-specific interventions, these findings can inform public health policies that promote lifelong healthy eating behaviors among Egyptian adolescents.

## Electronic supplementary material

Below is the link to the electronic supplementary material.

Supplementary Material 1

## Data Availability

The datasets used and/or analyzed for the current study are available from the corresponding author on reasonable request as excel sheet.

## Abbreviations

AOR: Adjusted odds ratio
ANLS: Adolescent nutrition literacy scale
BMI: Body mass index
CAPMS: Central agency for public mobilization and statistics
CI: Confidence interval
CNL: Critical nutrition literacy
COR: Crude odds ratios
FL: Food literacy
FNL: Functional nutrition literacy
INL: Interactive nutrition literacy
NL: Nutrition literacy
SD: Standard deviation
SFLQ: Short food literacy questionnaire
SPSS: Statistical package for the social sciences
TNL: Total nutrition literacy
WHO: World health organization
