# Correction: Nutrition literacy across adolescence stages in Egypt: a quartile-based analysis for tailored educational strategies

**DOI:** 10.1186/s12889-025-24422-4

**Published:** 2025-08-27

**Authors:** Iman H. Kamel, Ammal M. Metwally, Dina A. Zaki, Raefa Refaat Alam, Amani E. Ali, Ghada A. Elshaarawy, Safaa I. Abd El Hady, Ahmed G. Samy, Mona A. Elabd

**Affiliations:** 1 Child Health Department, Medical Research and Clinical Studies Institute, National Research Centre (Affiliation ID: 60014618), Dokki, Cairo Egypt; 2 Health and Community Medicine,Community Medicine Research Department, Medical Research and Clinical Studies Institute, National Research Centre (Affiliation ID: 60014618), Dokki, Cairo P.O. 12622 Egypt; 3 Faculty of Nursing, Mansoura National University, Al Dakhlyia, Egypt; 4 Technical Institute of Nursing, Sherbin, Al Dakhlyia Egypt; 5 Department of Psychiatry, Mansoura General Hospital, Ministry of Health and Population, El Dakahlyia, Egypt; 6 Biological Anthropology Department/ Medical Research and Clinical Studies Institute, National Research Centre (Affiliation ID: 60014618), Dokki, Cairo Egypt

**Correction: BMC Public Health 25**,** 2389 (2025)**.

**https://doi.org/10.1186/s12889-025-23583-6**

Following publication of the original article [1], the authors identified an error in the author name of Ahmed G. Samy.

The incorrect author name is: G. Samy.

The correct author name is: Ahmed G. Samy.

The author group has been updated above and the original article [1] has been corrected.
