# Supplementary material for: Nutrition literacy across adolescence stages in Egypt: a quartile-based analysis for tailored educational strategies
Source: BMC Public Health. 2025 Jul 5;25:2389. doi: 10.1186/s12889-025-23583-6 (PMC12228376; doi:10.1186/s12889-025-23583-6)
Supplement: Supplementary file 1 — Supplementary Material 1 [file 12889_2025_23583_MOESM1_ESM.doc]

**S Table-1: List of the randomly targeted households for the adolescence stage according to targeted governorates, locality, and sociodemographic status**

| **serial** | **Gov.** | **classification according to HDI** | **Kesm/Markaz** | **Urban** | **No surveyed** | **Rural** | | **No surveyed** |
| --- | --- | --- | --- | --- | --- | --- | --- | --- |
|  |  |  |  | **MCH/PHC Shiakha** |  | **Local Unit** | **MCH/PHC Village** |  |
| **1** | **Cairo** | **High** | **AlNozhah** | **Al Hicksit** | **75** |  |  |  |
|  |  | **middle** | **AlSaiedah Zainab** | **Alkabsh** | **75** |  |  |  |
|  |  | **low** | **AlSharabia** | **Al Amiria** | **75** |  |  |  |
| **2** | **Dakhlya** | **High** | **AlSenbelawin** | **Al Sinblaween city** | **45** | **Kafr Alruwk** | **Alshalaa** | **45** |
|  |  | **middle** | **MietSalsil** | **Mit salsil city** | **45** | **Alatihad** | **AlJafara** | **45** |
|  |  | **low** | **AlMataria** | **Almataria city** | **45** | **Alsafra** | **Al Dahear** | **45** |
| **3** | **Fayoum** | **High** | **Markaz of Al Fayoum** | **Alqism rabie** | **45** | **Dacia** | **Al Sunbat** | **45** |
|  |  | **middle** | **Markaz of**  **Senoures** | **Senoures** | **45** | **Terrsa** | **Alzawia El Khadra** | **45** |
|  |  | **Low** | **Markaz of Tamiaha** | **Tamiaha** | **45** | **Sarsna** | **Kafr Omira** | **45** |
| **4** | **Marsa Matrouh** | **High** | **Marsa Matrouh** | **MarsaMatrouh**  **(Alsanusia & Kilo 4)** | **45** | **Alkasr** | **Alkasr** | **45** |
|  |  | **middle** | **Al Hamam** | **Al Hamam City** | **45** | **Alsalam** | **Alsalam** | **45** |
|  |  | **Low** | **AlNajyla** | **AlNajyla** | **45** | **Almathany** | **Almathany** | **45** |
|  | **total** | **1035** |  |  | **630** |  |  | **405** |
